# Exploring the effects of aerobic and resistance exercise on mood-related symptoms and EEG activity

**DOI:** 10.3389/fnhum.2025.1562702

**Published:** 2025-03-28

**Authors:** Kihoon Yuk, Jawon Lim, Sangyun Kim, Tae Yeon Kim, Hyo Youl Moon

**Affiliations:** ^1^Department of Physical Education, College of Education, Seoul National University, Seoul, Republic of Korea; ^2^Institute of Aging, Seoul National University, Seoul, Republic of Korea; ^3^School of Biological Sciences, Seoul National University, Seoul, Republic of Korea

**Keywords:** aerobic exercise, resistance exercise, anxiety, depression, EEG

## Abstract

**Introduction:**

Anxiety and depression are psychiatric disorders that have a deleterious effect on human mental health. Meanwhile, various forms of exercise have been demonstrated to have beneficial effects on the reduction of symptoms of anxiety and depression. However, the disparate effects of each exercise on mood symptoms remain to be elucidated. This research examines the different effects of each type of exercise on mood symptoms and electroencephalography (EEG) activity.

**Methods:**

Accordingly, subjects engaged in six weeks of aerobic and resistance exercises with a 3-week washout period between each intervention. The Score of Hospital Anxiety and Depression Scale (HADS) was employed to assess the severity of both anxiety and depression. For the purposes of EEG analysis, we calculated the values of theta/beta ratio (TBR), Higuchi Fractal Dimension (HFD), and frontal alpha asymmetry (FAA).

**Results:**

Both types of exercise resulted in the alleviation of both anxiety and depression. Notably, aerobic exercise significantly decreased the score of HADS-A (anxiety), while resistance exercise significantly improved HADS-D (depression). In the context of EEG analysis, a significant decrease of TBR in the left frontal region was observed after aerobic exercise.

**Discussion:**

Therefore, these findings emphasize the importance of personalized exercise strategies for those suffering from anxiety and depression. Furthermore, further investigation into the impact of exercise on brain wave activities associated with anxiety and depression are needed.

## 1 Introduction

Anxiety and depression are mental illnesses that exhibit both similarities and differences (Demyttenaere and Heirman, 2020; Kalin, 2020). Symptoms such as abnormal excitement, dizziness, emotional arousal, and instability are associated with anxiety, whereas anhedonia, lack of energy, and low emotional response are linked to depression (Clark and Watson, 1991; Burns and Eidelson, 1998; American Psychiatric Association, 2013). A notably high prevalence of anxiety and depression among young adults has been recently observed (Barker et al., 2019; Goodwin, 2020). The elevated prevalence of anxiety and depression is a primary contributor to the observed increase in suicidal behaviors (Kalin, 2021). Therefore, it is imperative to develop and implement diverse strategies for the management of mental health concerns.

Recent studies have sought to find neurophysiological markers by measuring the brain activity in patients with anxiety or depression (Knyazev et al., 2005b; Lin et al., 2021; Liang et al., 2021). Among the various neuroimaging and neurophysiological techniques, electroencephalography (EEG) has been employed as a means of analyzing brain activity associated with anxiety and depression (Britton et al., 2016). EEG is conducted by attaching electrodes to the scalp, which allows for high accessibility and high temporal resolution (Song, 2016). EEG is particularly useful for identifying the electrical activity of the brain of various mental disorders (Yasin et al., 2021; Jaiswal et al., 2023). Recent studies have used EEG to compare the brain waves of patients with anxiety or depression with those of healthy controls to determine whether each group has a different level of activity in specific wave patterns (Knyazev et al., 2005a; Blackhart et al., 2006; Trambaiolli and Biazoli, 2020; Minkowski et al., 2021). One study suggested that a higher power spectrum value of alpha waves was observed in patients suffering from depression (Liang et al., 2021).

While various treatment methods are provided for the improvement of anxiety and depression, drug treatments, such as anxiolytic or antidepressant, are the most popular prescription for both diseases (Bandelow et al., 2015; Amare et al., 2017; Köhler et al., 2018). Despite anxiety and depression having different mechanisms (Boyer, 2000), antidepressants are dominantly prescribed for both anxiety and depression. However, these common treatments can be problematic. While anxiety requires immediate symptom relief through anxiolytics, antidepressants require minimum 2 weeks to show their effects (Zoberi and Alec Pollard, 2010). Moreover, antidepressants may carry a risk of excessive physiological reactions and induce additional anxiety as side effects (Ballew, 2021; Fayez and Gupta, 2023). The current situation that antidepressants are prescribed for anxiety treatment despite danger of side effects reveals the lack of accurately established disorder-specific strategies.

Considering that drug treatment has critical side effects (Ramic et al., 2020), a demand for treatment with fewer side effects is increasing. Exercise has been proposed as an effective therapeutic tool for improving mental illness and neurodegenerative conditions (Hearing et al., 2016; Quan et al., 2020). A substantial body of evidence from animal and human studies has demonstrated that exercise effectively increases neurotransmitters, growth factors, blood flow formation, and neurogenesis, ultimately leading to improvements in brain function (Voss et al., 2013; Ryan and Kelly, 2016; Moon et al., 2019). In conclusion, a multifaceted approach may be needed to address each mood.

Long-term exercise has been demonstrated to have beneficial effects on both mood and the symptom relief, as well as on EEG changes (Liang et al., 2021). A number of reviews have examined whether the EEG pattern of patients are altered through exercise interventions (Gramkow et al., 2020; Hosang et al., 2022). However, although previous studies have primarily focused on comparing the effects of aerobic and resistance exercise on anxiety and depression, either separately or in relation to each other, no research has simultaneously addressed all four aspects; comparing which types of exercise are more beneficial for each mood. Furthermore, there is a paucity of studies investigating these effects alongside with changes in EEG patterns. In a previous pilot study, we posited that 6 weeks of aerobic or resistance exercise may result in different patterns of anxiety and depression alleviation and distinct EEG alterations (Yuk et al., 2024). Specifically, 6 weeks of resistance exercise significantly reduced depression-related symptoms, whereas aerobic exercise reduced anxiety-related symptoms. Thus, we would like to propose the possibility that different chronic exercise modalities may have different effects; determining which exercise is more effective for mood-related EEG changes.

In light of these findings, further research is required to gain a deeper understanding of the relationship between changes in brain wave levels and the manifestation of anxiety and depression symptoms. Specifically, this was a secondary study based on the pilot study, which examined the effects of two types of exercise on anxiety and depression symptoms (Yuk et al., 2024). The aim of this study is comparing the effects of resistance and aerobic exercise for the alleviation on mood-related symptoms. We hypothesized that each exercise would play a different role in improving anxiety and depression: aerobic exercise would alleviate anxiety and resistance exercise would reduce depression.

## 2 Materials and methods

### 2.1 Ethic declarations

All experimental procedures were approved by the Institutional Review Board of Seoul National University in accordance with the standards of the Declaration of Helsinki of the World Medical Association (IRB No. 2309/004-010). Prior to the experiment, written consent and questionnaire scores (online) were obtained from all participants. All subjects were informed about the procedures and purpose of the study, as well as the potential risks of exercise protocols in both oral and written forms, and they confirmed their willingness to participate.

### 2.2 Participants

Healthy adults aged 19–39 years were recruited from Seoul National University in response to online/offline advertisements. Participants taking anxiolytics/antidepressants or participating in regular exercise were excluded. Participants were randomly divided into three groups with one control (non-exercise) group (CTL) and two experimental groups.

### 2.3 Study design and procedures

The overall procedure of this study started in September 2023 and was completed in April 2024. Initially, 30 healthy participants (age: 26.32 ± 4.58) volunteered.

**Figure 1:**
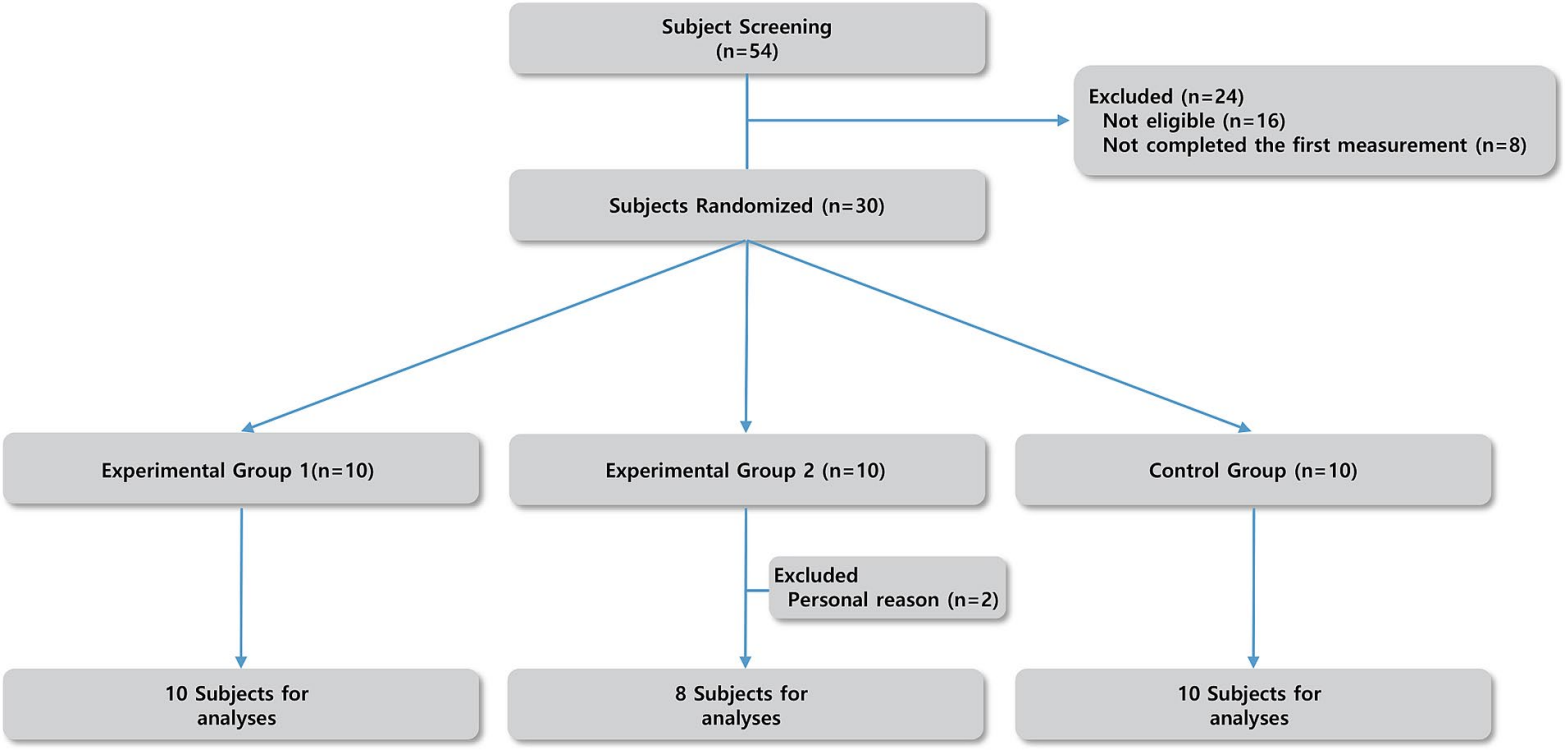
Flow diagram of the study design.

The flow diagram of this study is illustrated in Figure 1. Exercise groups performed two 6-week exercise sessions twice a week: a total twelve-week with a 3-week washout (O’Donnell et al., 2011; Sanghuachang et al., 2023) session after one exercise session (Supplementary Figure S1). All interventions were taken by the same researcher and same environment (indoor lab).

Participants in experimental groups attended a total of 24 sessions according to the experiment schedule. The experiment consisted of the following phases: Exercise 1 (Week 1–6) / Wash-out (Week 7–9) / Exercise 2 (Week 10–15) (Supplementary Figure S1).

At the first visit (screening), the Physical Activity Readiness Questionnaire (PAR-Q), International Physical Activity Questionnaire (IPAQ), and the score of Hospital Anxiety and Depression Scale (HADS) were measured. Assessment was done before the first exercise session in Week 1 and 10 and after the last exercise session in Week 6 and 15 (Supplementary Figure S1). The assessment measures include body composition, resting heart rate, HADS, and 10-channel EEG data.

### 2.4 Exercise procedures

#### 2.4.1 Aerobic exercise program

The aerobic exercise program was conducted based on ACSM guidelines (Pescatello, 2014). Exercise was done on a cycle ergometer (Monark 829E, Sweden). The individual VO₂max value was predicted through a 6-min Åstrand cycle ergometer test (Åstrand and Ryhming, 1954). Each session consisted of a 3-min warm-up and cool-down of light intensity activity (without resistance load). After warm-up, the cycling exercise was done at a moderate intensity [i.e., 60%~70% of individual heart rate reserve (HRR)] lasting 30 min. The exercise intensity was gradually increased based on individual’s heart rate increase. The individual’s heart rate was tracked throughout each session using Apple Watch SE (Apple, United States).

#### 2.4.2 Resistance exercise program

The resistance exercise program was conducted based on ACSM guidelines and previous literature (Pescatello, 2014; LeBouthillier and Asmundson, 2017; O'Sullivan et al., 2023). The specific contents are summarized in Supplementary Table S1. Adaptation session was provided for participants for 30 min prior to exercise sessions (resistance). Participants performed 3 sets of 10–12 repetitions for each movement, with 60–90 s of time intervals between each set (Willardson, 2006). The intervention weight was recorded and progressively increased based on the participants’ adaptation. All human-human interactions were controlled.

### 2.5 Physical and mood symptom measurements

Weight, body fat percentage, and skeletal muscle mass of participants were measured by InBody 770 (InBody, Korea). During the measurement, the participants were instructed to remove accessories or metallic substances, including socks, earrings, and necklaces. Resting heart rate was measured using Apple Watch SE (Apple, United States) (Wallen et al., 2016; Falter et al., 2019). Blood pressure was measured using BPB10750 (InBody, Korea).

The HADS is a questionnaire composed of total of 42 points, divided into 21 points (anxiety/depression) and maximum 3 points per question (7 questions each), which is used to evaluate the severity of anxiety and depressive symptoms. The Korean version of the HADS was used (Min et al., 1999). The severity of each mood according to HADS scores are as represented: 0–7 normal, 8–10 borderline abnormal, 11–21 abnormal. HADS has a high validity and sensitivity on both anxiety (*ɑ* = 0.89) and depression (*ɑ* = 0.86) (Oh et al., 1999).

### 2.6 EEG analysis

EEG measurements were conducted in a dark, quiet condition to make subjects maintain resting state. After a short adaptation period, subjects were instructed to close their eyes and minimize all movements including eye movements. We provided the subjects a pair of earplugs to minimize external noises. EEG was recorded for 3 min using QEEG-64FX (Laxtha Inc., Korea) (Mahato et al., 2020; Lim et al., 2018). The whole recording was done in accordance with the 10–20 system using 10 channels (Klem et al., 1999). Electrodes were attached to Fp1, Fp2 (prefrontal), F3, F4 (frontal), T7, T8 (temporal), P3, P4 (parietal), O1, and O2 (occipital). The right and left earlobe each served as the ground (GND) and reference load (A2). The sampling rate was 250 Hz/channel, with a 12-bit A/D converter.

After raw data was extracted, it was filtered with a 0.5 Hz low-pass filter, a 50 Hz high-pass filter, and a notch filter set at 60 Hz to eliminate additional movements or electrical noises (Mahato et al., 2020), and was processed with Savitzky–Golay filter (window length: 51) to remove noise artifacts (Al-Qazzaz et al., 2019) using Telescan program (Ver. 3.29., Laxtha Inc. Korea). EEG signals were assessed by three individuals who were blinded to the experiment. Visual inspection was done to exclude signals contaminated by external noise or other artifacts.

Each set of EEG data was analyzed according to previous studies that suggested the relationship between each biomarker and state of anxiety or depression. We set the frequency of each wave: beta (4–7.99 Hz), alpha (8–12.99 Hz), and theta (13–29.99 Hz). As anxiety and depression biomarkers, we calculated the value of the theta/beta ratio (TBR) (Wei et al., 2020; Chang and Choi, 2023), Higuchi Fractal Dimension (HFD) values of each brain region (Bachmann et al., 2013), and frontal alpha asymmetry (FAA) (Seok et al., 2007). HFD values were calculated using Python v3.12.

### 2.7 Statistical analysis

Statistical analysis was performed using Graph Pad Prism V.10.2.2 (Graph Pad Software Inc., CA, United States) and R (version 4.4.2, R Foundation for Statistical Computing, Vienna, Austria). All diagrams and data are presented as mean ± standard error of the mean (SEM). Data analyses were performed based on three groups according to exercise type [CTL, Aerobic (AE), Resistance (RE)].

The normality of data was assessed through Shapiro–Wilk test. An unpaired t-test or Wilcoxon rank sum test was performed to compare baseline values between groups. A paired t-test was performed to compare the exercise effect after each intervention. A one-way ANOVA was conducted to compare the average score change deviation within each group of participants. A two-way ANOVA with repeated measurements was used to compare the effect of exercise after intervention. A Bonferroni *post-hoc* test was applied to verify differences within the group. Values of *p* < 0.05 were considered statistically significant. Effect size (ES) was calculated based on the statistical analysis method. The ES of t-test was measured as Cohen’s d, while the ES of two-way with repeated measurements was measured as partial eta squared ( $\eta_{p}^{2}$ ).

## 3 Results

### 3.1 Participants

In total, 18 participants (female 83.3%) composed the experimental group and 10 participants (female 70%) belonged to the control group. During the experiment, two female participants from experimental group 2 dropped out during the washout session; however, their data from the resistance exercise session was included in the analysis as both participants had completed the entire resistance exercise session.

No significant differences were observed in the baseline values of questionnaire scores of HADS-A (anxiety), HADS-D (depression), and the average Metabolic Equivalent Task (MET) value according to IPAQ between the experimental and control groups (Table 1).

**Table 1:** Baseline characteristics of participants.

Mean ± SEM	Exercise (*n* = 18)	Control (*n* = 10)	*p*-value	Effect size (d)
Height (cm)	164.63 ± 0.31	163.69 ± 0.58	0.6893	0.16
Age (yrs)^†^	26.83 ± 0.29	25.3 ± 0.31	0.5454	0.33
HADS-A (points)^†^	10.89 ± 0.82	10 ± 0.99	0.7306	0.26
HADS-D (points)^†^	10.17 ± 0.88	9.6 ± 0.69	0.6656	0.17
IPAQ (METs)	731.53 ± 176.88	922.65 ± 285.13	0.5955	0.24

No significance was observed between groups. Statistical analysis was performed using an unpaired t-test or Wilcoxon rank sum test. HADS-A, Hospital Anxiety and Depression Scale-Anxiety; HADS-D, Hospital Anxiety and Depression Scale-Depression; IPAQ, International Physical Activity Questionnaire; ^†^Wilcoxon rank sum test.

### 3.2 Physical measurements

Table 2 presents the changes in physical characteristics observed following each intervention. No significant changes were noted within groups throughout the intervention period in resting heart rate, blood pressure, and body fat percentage following aerobic exercise. However, only resistance exercise resulted in a significant increase in body weight (*p* = 0.033, ES = 0.15) and skeletal muscle mass (*p* = 0.039, ES = 0.02) compared to the baseline measurement.

**Table 2:** Physical characteristics of participants.

	CTL (*n* = 10)		AE (*n* = 16)		RE (*n* = 18)		
	M	SEM	M	SEM	M	SEM	Effect size ( $\eta_{p}^{2}$ )
Heart rate (bpm)							
Before (Baseline)	78.2	2.48	77.38	2.3	76.33	1.85	0.1
After	74.8	2.02	74.38	2.12	79.11	2.53	
Systolic blood pressure (mmHg)							
Before (Baseline)	109.8	2.75	111.25	2.69	111.22	2.69	0.006
After	110	2.47	112	2.54	110.44	1.68	
Diastolic blood pressure (mmHg)							
Before (Baseline)	64.7	2.89	70.25	2.42	70.78	2.83	0.04
After	67	2.29	69.5	3.14	66.61	2.13	
Body weight (kg)							
Before (Baseline)	62.44	4.6	59.25	2.32	58.14	2.27	0.15
After	62.43	4.31	58.8	2.26	**58.73** ^*^	2.26	
Body fat percentage (%)							
Before (Baseline)	28.48	2.64	26.2	1.63	26.07	1.41	0.02
After	28.29	2.5	25.49	1.61	25.88	1.51	
Skeletal muscle mass (kg)							
Before (Baseline)	24.1	2.01	23.82	0.99	23.43	0.98	0.03
After	24.24	1.94	23.91	1.05	**23.77** ^*^	1.04	

A significant increase in body weight and skeletal muscle mass was observed. Statistical analysis was performed using a two-way ANOVA with Bonferroni *post-hoc* test. (^*^p < 0.05).

### 3.3 Mood symptom measurements

**Figure 2:**
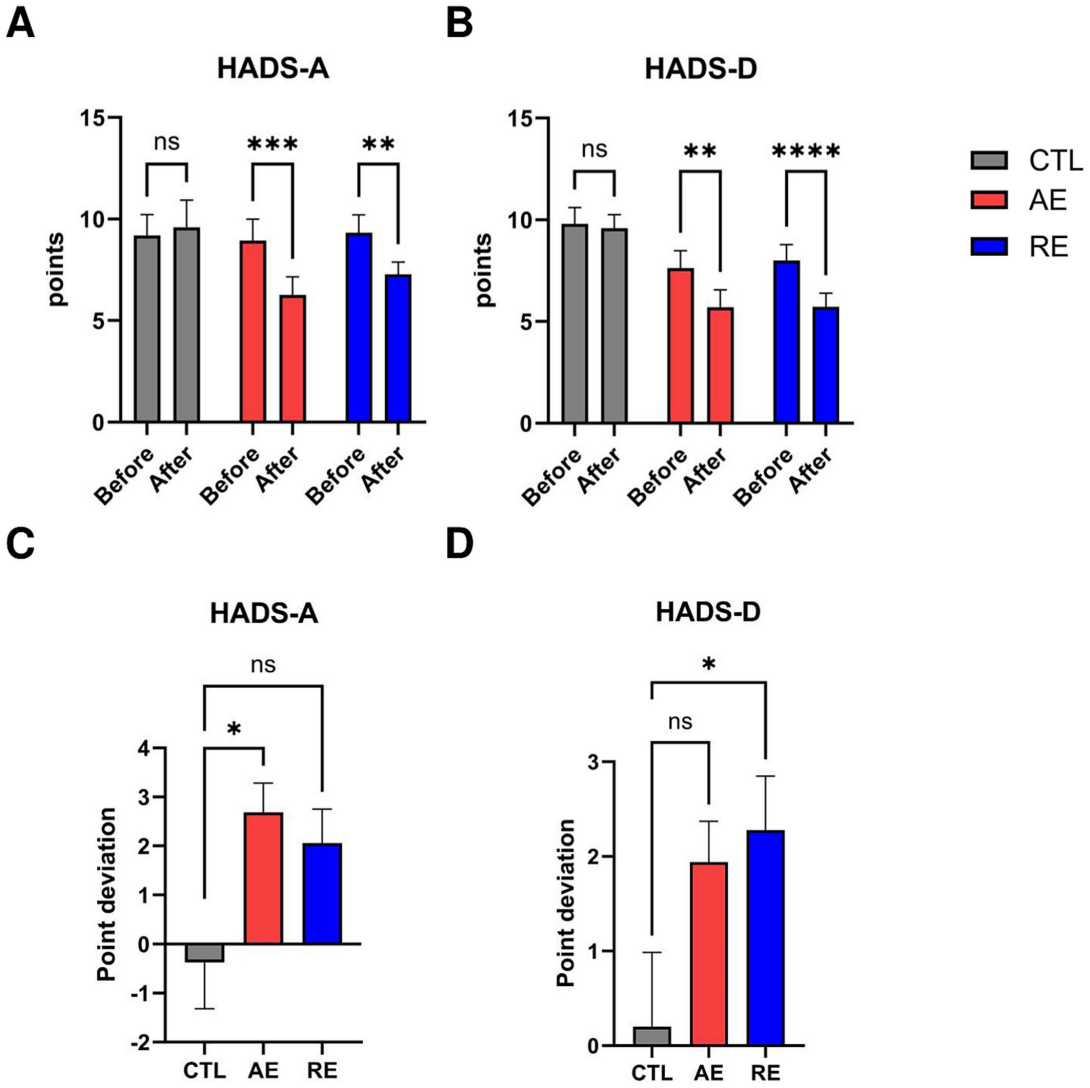
Changes in questionnaire scores after exercise. **(A)** HADS-A score, **(B)** HADS-D score, **(C)** Point deviation of HADS-A, **(D)** Point deviation of HADS-D. CTL (*n* = 10), AE (*n* = 16), and RE (*n* = 18). Statistical analysis was performed using two-way ANOVA with Bonferroni *post-hoc* test. Data was presented as mean ± SEM (^*^p < 0.05, ^**^p < 0.01, ^***^p < 0.001, ^****^p < 0.0001, ns = not significant). HADS-A, Hospital Anxiety and Depression Scale-Anxiety; HADS-D, Hospital Anxiety and Depression Scale-Depression; CTL, Control; AE, Aerobic exercise; RE, Resistance exercise.

We measured the score of HADS to evaluate the changes in anxiety and depression symptoms. A two-way ANOVA on HADS-A scores revealed a significant main effect from group by time interaction *F*_(2,41)_ = 4.113, *p* = 0.024, ES = 0.17. A *post-hoc* analysis revealed a significant decrease in HADS-A score following both AE (*p* = 0.0003) and RE (*p* = 0.003) (Figure 2A). There was no significance in within the CTL group (*p* = 0.647).

There was a tendency toward a group by time interaction *F*_(2,41)_ = 3.011, *p* = 0.06, ES = 0.13 was observed on HADS-D scores. A significant reduction in HADS-D scores was observed following both AE (*p* = 0.011) and RE (*p* < 0.0001). There was no significance in the CTL group (*p* = 0.776) (Figure 2B). The results demonstrated that both 6 weeks of aerobic and resistance exercise caused a significant decrease in anxiety-related scores and depression-related scores.

To ascertain which exercise exerts a more pronounced effect on the reduction of anxiety and depressive symptoms, we conducted a comparative analysis of the mean score change deviation within each participant group. The results showed that aerobic exercise resulted in a statistically significant reduction in anxiety scores compared to the CTL group (*p* = 0.025) (Figure 2C). Conversely, resistance exercise led to a significant decrease in depression scores compared to the CTL group (*p* = 0.044) (Figure 2D). In conclusion, both exercises exhibited a notable impact on mood symptoms, with aerobic exercise particularly contributing to anxiety improvements and resistance exercise to depression relief.

### 3.4 EEG analysis

Prior research indicates that the severity of anxiety may be indicated by TBR of the frontal region and FAA (Seok et al., 2007; Al-Ezzi et al., 2020). Conversely, TBR of the temporal region, HFD, and FAA may be indicative of depression-related EEG activity, as previously observed in studies (Seok et al., 2007; Bachmann et al., 2013; Ren et al., 2020).

**Figure 3:**
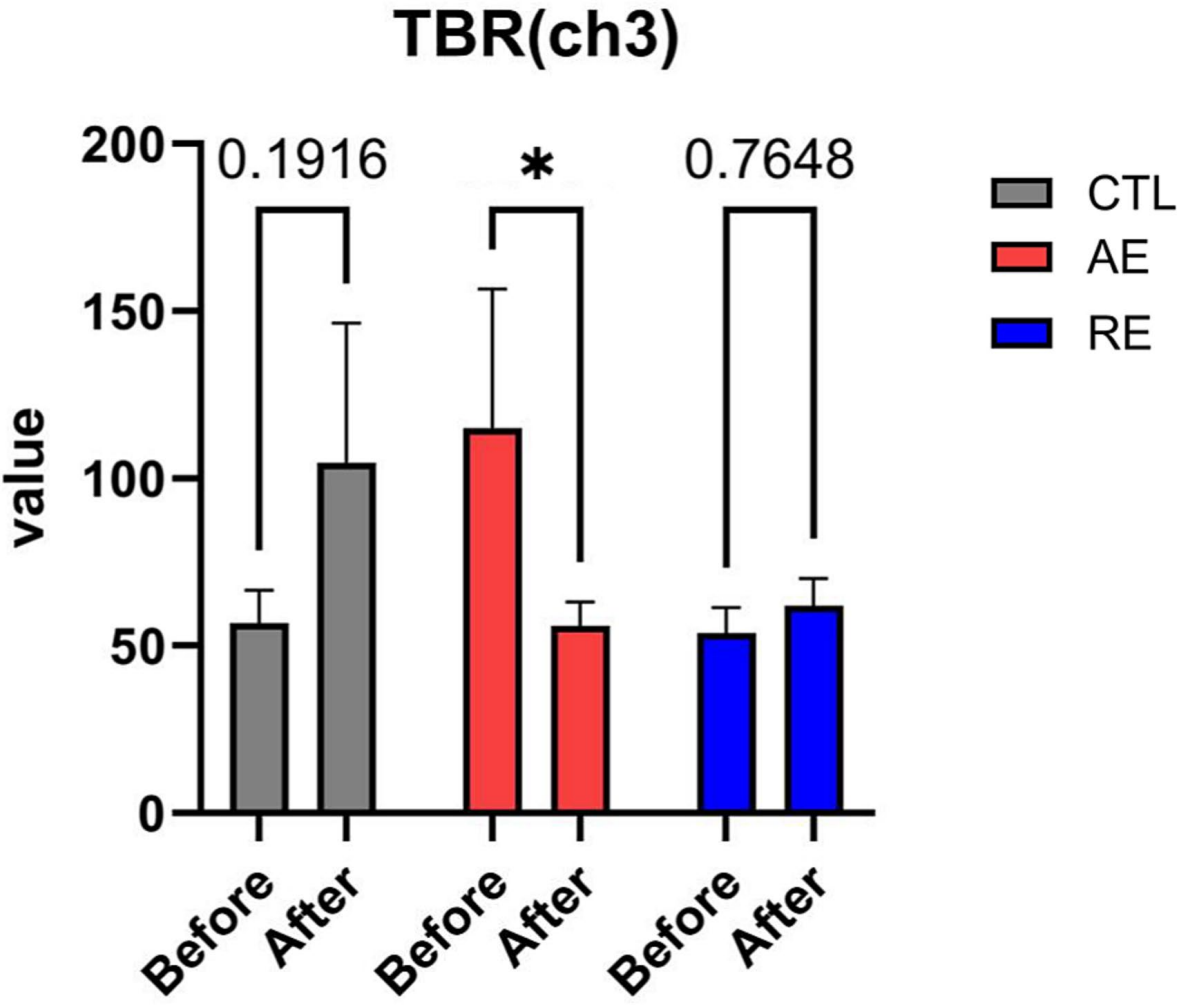
Changes of anxiety-related EEG patterns after exercise. TBR (ch3). CTL (*n* = 10), AE (*n* = 16), and RE (*n* = 18). Statistical analysis was performed using two-way ANOVA with Bonferroni *post-hoc* test. Data was presented as mean ± SEM (^*^p < 0.05, ns = not significant). TBR, Theta/beta ratio; CTL, Control; AE, Aerobic exercise; RE, Resistance exercise.

**Figure 4:**
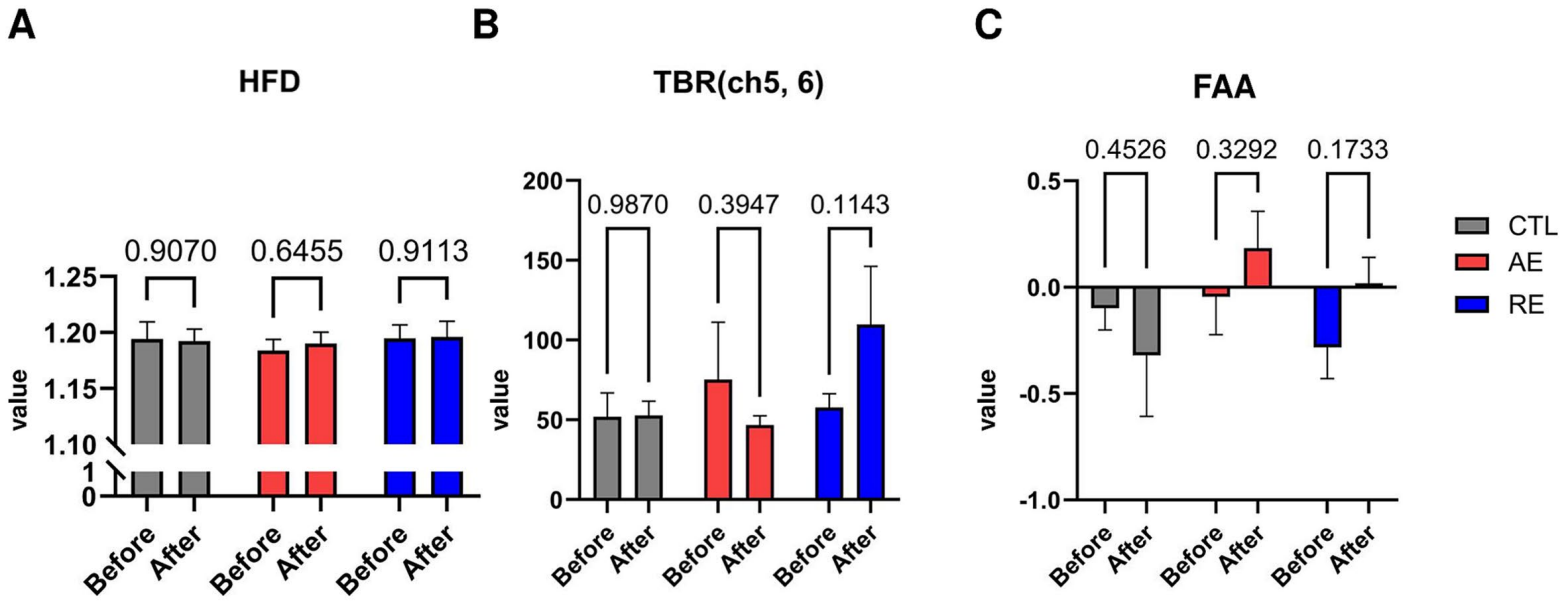
Changes of depression-related EEG patterns after exercise. **(A)** HFD, **(B)** TBR (ch5, 6), **(C)** FAA. CTL (*n* = 10), AE (*n* = 16), and RE (*n* = 18). Statistical analysis was performed using two-way ANOVA with Bonferroni *post-hoc* test. Data was presented as mean ± SEM (^*^p < 0.05, ns = not significant). HFD, Higuchi Fractal Dimension; TBR, Theta/beta ratio; FAA, Frontal alpha asymmetry; CTL, Control; AE, Aerobic exercise; RE, Resistance exercise.

While no significant differences were observed in the TBR of frontal region between the RE and CTL groups, a notable decrease of TBR in the left frontal region (ch3) following exercise was verified (*p* = 0.04, ES = 0.1) (Figure 3). No significant alterations in HFD (ES = 0.004), TBR of the temporal region (ES = 0.07), and FAA were discerned (ES = 0.05) (Figures 4A–C). These finding imply that aerobic exercise may influence EEG patterns, particularly the ratio of theta and beta waves in the frontal region. However, no notable changes were observed following resistance exercise.

## 4 Discussion

A substantial body of evidence from both non-clinical and clinical studies indicates the potential to differentiate between anxiety and depression at the mechanistic level (Renner et al., 2018) and to apply different therapeutic strategies for each mood state (Yuk et al., 2023). With regard to the findings of our pilot trial, the objective was to find the disparate effects of aerobic and resistance exercise on the reduction of anxiety/depression-related symptoms and the alteration of brain wave patterns. We observed a reduction in anxiety symptoms after aerobic exercise, while a reduction in depressive symptoms was observed after resistance exercise. We were also able to confirm EEG pattern changes associated with anxiety symptoms. These findings provide valuable insights for promoting exercise for the enhancement of mental health, especially applying individualized exercise strategies according to one’s mental status.

Firstly, with regard to the body composition results, 6 weeks of resistance exercise resulted in a significant increase in skeletal muscle mass and body weight. Nevertheless, no significant differences were observed after aerobic exercise. Given that our aerobic exercise regimen consisted of 2 days of cycling at moderate intensity, it is plausible that a higher frequency or intensity may be required to induce significant alterations in body composition (Mendes et al., 2013; Bryner et al., 1997). Notwithstanding, the Åstrand test results demonstrated a significant increase in VO₂max among all participants following aerobic exercise (Supplementary Figure S2A), which corroborates the findings of a previous study (Mendes et al., 2013). In addition, the relative load calculation results exhibited a notable increase in load throughout the resistance session (Supplementary Figure S2B). This suggests that 6 weeks of resistance exercise may effectively enhance skeletal muscle mass, consistent with the previous finding that a 6–12 repetition maximum (RM) may facilitate muscle hypertrophy (Kraemer and Ratamess, 2004). Our findings indicate that 6 weeks of aerobic exercise is effective for elevating cardiorespiratory fitness ability, while resistance exercise may promote muscle mass gain.

Secondly, changes in the questionnaire scores were observed in order to investigate the changes in anxiety and depressive symptoms. Consequently, both types of exercise resulted in a reduction in the severity of both anxiety and depression, as indicated by HADS criteria, from mild to normal levels. In accordance with our findings, previous studies have highlighted that exercise has a beneficial impact on human mental health (Fetzner and Asmundson, 2015; LeBouthillier and Asmundson, 2017; O'Sullivan et al., 2023). It is noteworthy that the comparison of questionnaire score deviations for each exercise revealed that each exercise can specifically target the alleviation of different mood symptoms. While several studies support that aerobic exercise effectively reduces anxiety symptoms (Fetzner and Asmundson, 2015; Hill et al., 2019) and resistance exercise contributes to a decrease in depression symptoms (Zhao et al., 2020), our results suggest that the effects of different types of exercise may be differentiated. Specifically, 6 weeks of aerobic and resistance exercises (once per week) have been shown to, respectively, reduce BAI and BDI scores (Yuk et al., 2024). Thus, participating in 6 weeks of moderate aerobic and resistance exercise has the potential to offer comprehensive benefits with regards to mental health enhancement. However, participating in different types of exercise based on one’s current mood symptoms, aerobic exercise for anxiety-like symptoms and resistance exercise for depression-like symptoms can be a more efficacious approach.

Next, we investigated the EEG pattern changes according to previous literature, which confirmed the difference between anxiety or depression patients and healthy controls. In light of the notable decline in frontal TBR, recent studies have indicated a positive correlation between TBR and anxiety level scores (Putman et al., 2014; Al-Ezzi et al., 2020). Therefore, when considering the alterations in questionnaire scores and outcomes of TBR, it can be postulated that aerobic exercise may serve to mitigate anxiety symptoms and EEG patterns. Moreover, given that our subjects were not clinically diagnosed as patients, our findings may imply that frontal TBR functions as an anxiety-specific biomarker for individuals without a clinical diagnosis of anxiety disorders. Notably, aerobic exercise may significantly alter.

Previous studies have shown that individuals diagnosed with major depressive disorder exhibited elevated HFD values (Bachmann et al., 2013; de Aguiar Neto and Rosa, 2019) and alterations in theta oscillatory activity (Ren et al., 2020; Chang and Choi, 2023). Accordingly, we initially assessed the values of HFD and the TBR of the temporal region as potential depression-specific biomarkers. However, the results revealed that the implementation of 6 weeks of aerobic and resistance exercises did not cause a discernible alteration in the values of depression-specific biomarkers across all groups. While some studies question whether HFD can explain the depression severity (Ahmadlou et al., 2012; Lord and Allen, 2023), the majority of previous studies observed significant higher HFD from patients diagnosed with depression compared to that of healthy controls (Akar et al., 2015a; Akar et al., 2015b). As our study participants did not have any clinical diagnosis of mood disorders, we postulated that HFD may serve as a biomarker only when the symptom severity of an individual exceeds a certain level sufficient to be diagnosed as a disease; the severity of the participants was not sufficient to detect HFD differences. Nonetheless, further studies are required to elucidate the relationship between exercise and HFD is needed. With regard to temporal TBR, our findings were consistent with a study that did not find a significant difference between depressed patients and healthy controls (Chang and Choi, 2023). This is the first study to confirm the exercise effect on depression through HFD and temporal TBR. However, the effects of each exercise on these two EEG biomarkers remain unclear.

Another mood-related biomarker we measured was FAA. The FAA serves as a biomarker indicative of disparate activities within the left and right frontal cortex. In particular, increased alpha absolute power in the left hemisphere is indicative of a lack of approach behavior, which is common phenomenon observed in patients with depression (de Aguiar Neto and Rosa, 2019). However, this may be reversed by increasing anxiety severity (Seok et al., 2007). Throughout our trial, we did not observe a significant change in FAA after each intervention. These results were similar to those of our pilot study, which also did not observe any significance after both types of exercise. There are numerous studies examining the changes in FAA together with depression (Bond et al., 2018; Hong et al., 2020); however, the results explaining how exercise regulates the asymmetry patterns are yet contradictory (Reid et al., 1998; Hong et al., 2020). One meta-analysis study questioned the validity of FAA, since patterns of FAA varied according to age or sex, and a single variable that explains the relationship between FAA and depression was not found (Van Der Vinne et al., 2017). Therefore, while some studies utilize FAA as a representative biomarker to explain depression symptoms, further studies are needed to precisely organize the relationship between FAA and mood disorders, and to clarify the effect of exercise on FAA as well.

Our study has some limitations. Firstly, the program G-Power 3.1 (Heinrich-Heine-Universität Düsseldorf, Germany) recommended to recruit total of 30 participants (assumed as ‘ANOVA: Repeated measures, within-between interaction,’ effect size = 0.25, power = 0.8, alpha error probability = 0.05). However, due to dropouts, the final sample size was smaller than the recommended number. Since the G-Power *post-hoc* power analysis suggests the statistical power of this study as 0.6 (assumed as “ANOVA: Repeated measures, within-between interaction,” effect size = 0.25, total sample size = 28, alpha error probability = 0.05), future research conducted with a larger sample size (*N* = 42 for power 0.8) could display meaningful results. Second, the absence of objective biomarkers precludes the determination of whether the designated washout period was sufficient to eliminate the effects of exercise on mood. A 3-week wash-out period was implemented, based on the findings of a previous study which suggested that 3-week washout may eliminate exercise effects (Otsuka et al., 2021). Although our study did not reveal any significant discrepancies in the baseline scores of Week 1 and Week 10 (Supplementary Figure S3), the necessity for a precise biomarker to demonstrate the wash-out period remains. Thirdly, we did not control confounding lifestyle variables, such as diet, sleep patterns, etc. Lastly, we had technical limitations regarding EEG analysis: measuring and controlling the impedance was difficult using a dry cap. Moreover, additional artifacts such as signal noise, movement, etc. may have influenced the results. These limitations highlight the need for further studies to address these issues.

In conclusion, both types of exercise have been shown to help reduce symptoms associated with anxiety and depression. Nevertheless, moderate aerobic exercise may be an effective method of reducing anxiety symptoms, whereas moderate resistance exercise has been shown to be more effective in reducing depressive symptoms. The results of the EEG analysis results indicated that aerobic exercise may contribute to a tendency for decrease in TBR (ch3, 4) and a significant reduction in TBR (ch3). However, there were no significant changes in, HFD, and FAA. These findings suggest the possibility that different types of exercise may alleviate different mood symptoms and also lead to changes in mood EEG biomarkers. To the best of our knowledge, this is the first study to directly compare two types of exercise in terms of mood symptoms and EEG pattern changes.

## Data Availability

The original contributions presented in the study are included in the article/Supplementary material, further inquiries can be directed to the corresponding author.
