# Supplementary material for: Exploring the effects of aerobic and resistance exercise on mood-related symptoms and EEG activity
Source: Front Hum Neurosci. 2025 Mar 28;19:1562702. doi: 10.3389/fnhum.2025.1562702 (PMC11985855; doi:10.3389/fnhum.2025.1562702)
Supplement: Supplementary file 1 [file Table_1.docx]

**Supplementary Information**

Supplementary Figure S1. Schematic diagram of the research design.

Supplementary Figure S2. (A) Changes of VO₂max according to Åstrand test (n = 16). (B) Changes of relative load after resistance exercise (n = 18). Statistical analysis was performed using a paired t-test (** p < 0.01, **** p < 0.0001). Data was presented as mean ± SEM.

Supplementary Figure S3. Comparison of baseline (Week 1 and Week 10) scores of EX 1 and EX 2. (A) HADS-A (EX 1), (B) HADS-A (EX 2), (C) HADS-D (EX 1), (D) HADS-D (EX 2). EX 1 (n = 9), EX 2 (n = 9). Statistical analysis was performed using a paired t-test. Data was presented as mean ± SEM. EX 1; Experimental group 1. EX 2; Experimental group 2. HADS-A; Hospital Anxiety and Depression Scale-Anxiety. HADS-D; Hospital Anxiety and Depression Scale-Depression.

Supplementary Table S1. Resistance exercise program.

Supplementary Table S1.

| **Program 1** | **Program 2** |
| --- | --- |
| Abdominal crunches | Plank |
| Machine leg press | Machine leg extension |
| Machine leg curl | V squat |
| Dumbbell row | Cable row |
| Machine chest press | Dumbbell bench press |
| Lat pull down | Inverted row |
| Machine Shoulder press | Dumbbell Shoulder press |
