# Supplementary figures and images for: Exploring the effects of aerobic and resistance exercise on mood-related symptoms and EEG activity
Source: Front Hum Neurosci. 2025 Mar 28;19:1562702. doi: 10.3389/fnhum.2025.1562702 (PMC11985855; doi:10.3389/fnhum.2025.1562702)

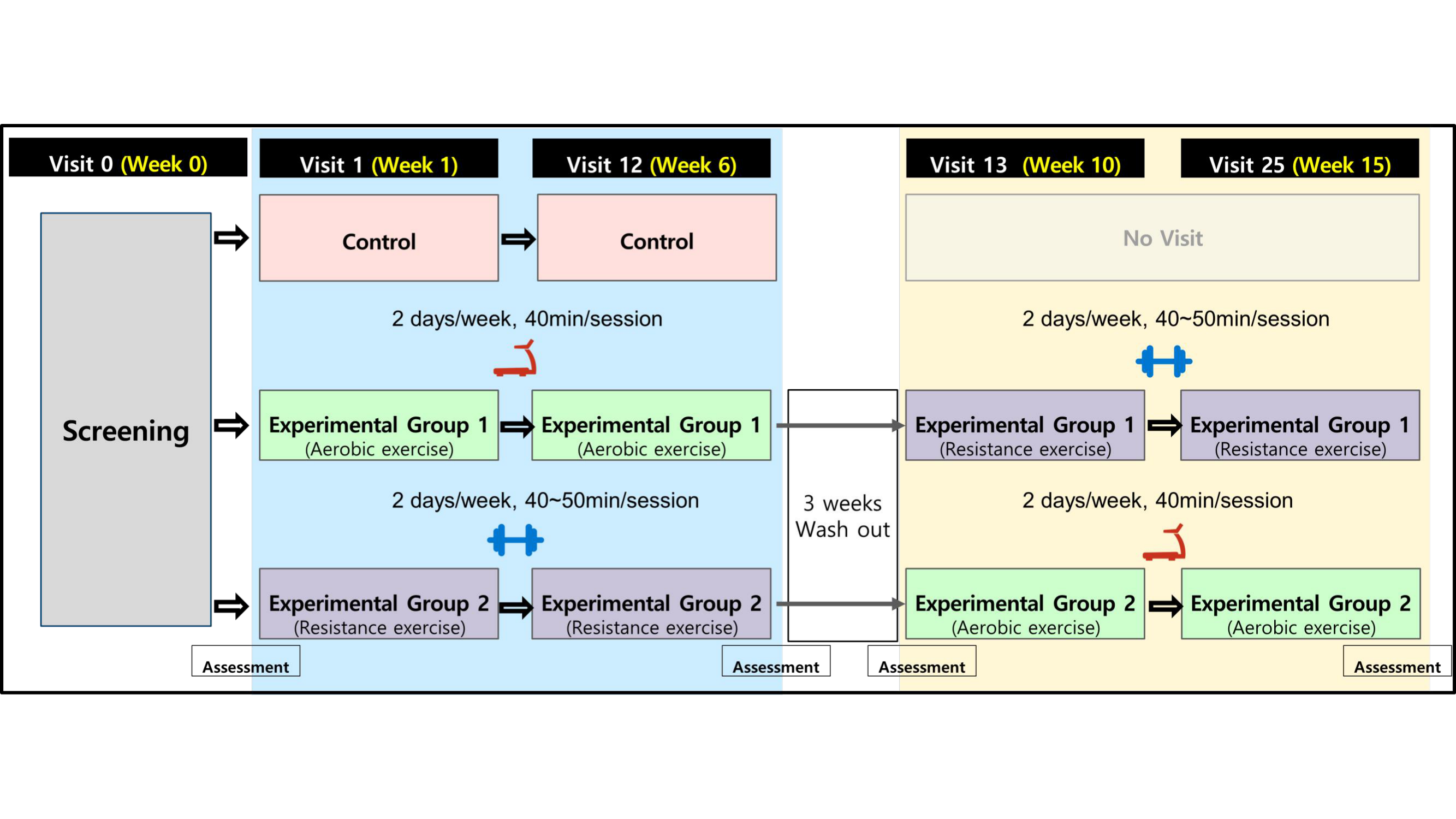

Supplement: Supplementary file 2 [file Image_1.tiff]

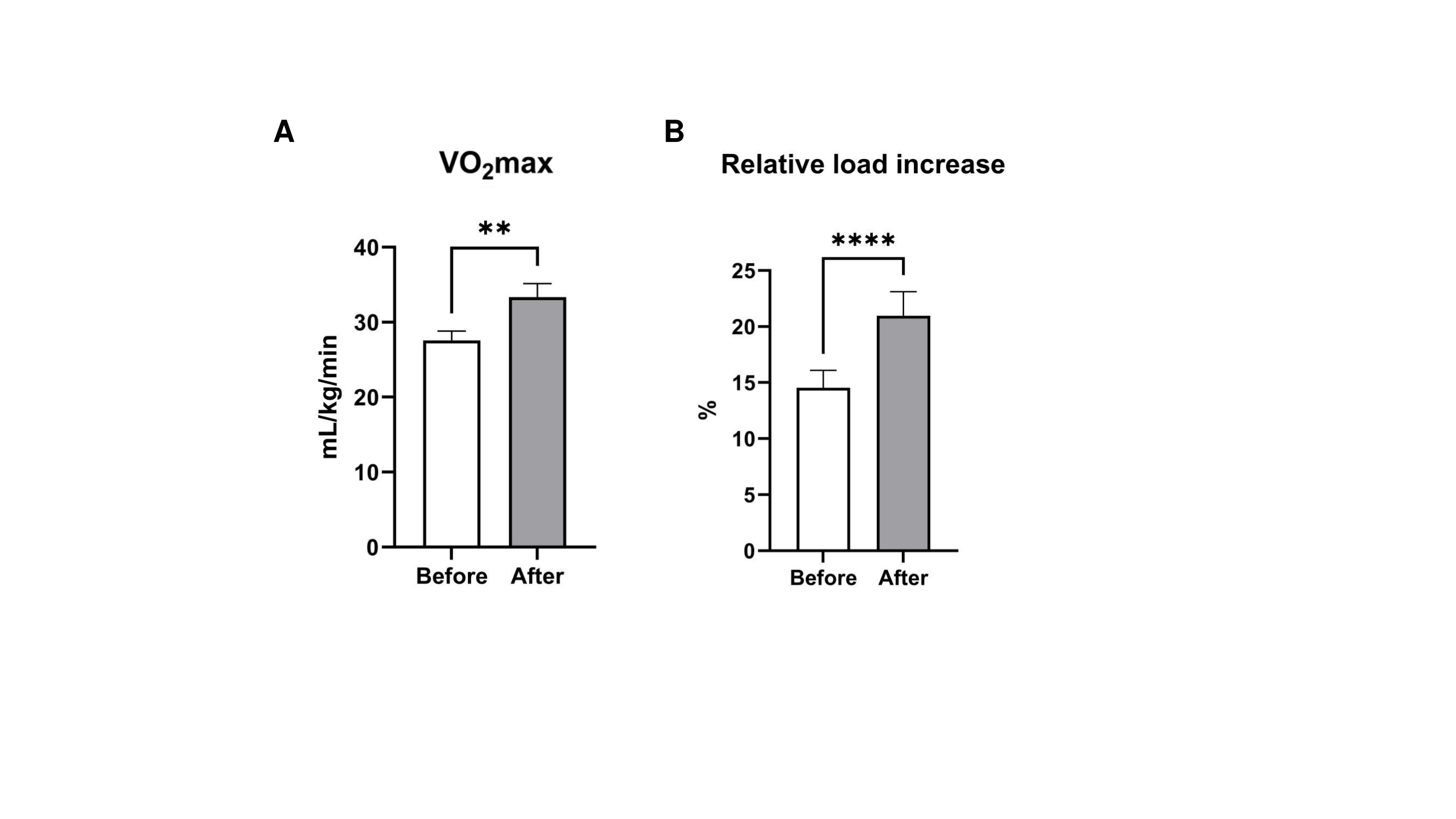

Supplement: Supplementary file 3 [file Image_2.tiff]

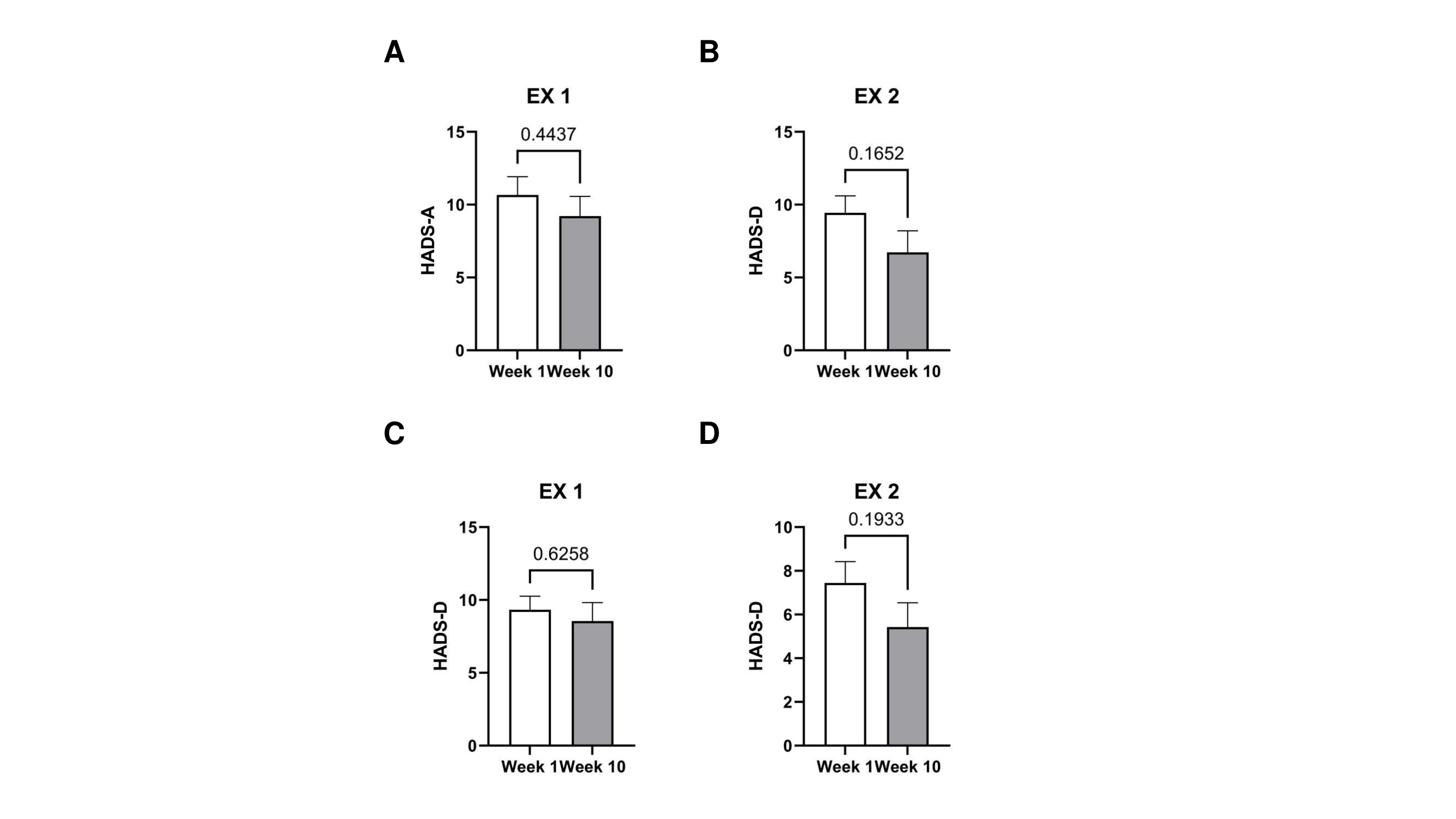

Supplement: Supplementary file 4 [file Image_3.tiff]
